# The Association Between Home‐Visit Nursing Use and the Incidence of Potentially Avoidable Hospitalisation and Its Duration Among Community‐Dwelling Older People: A 12‐Month Prospective Cohort Study

**DOI:** 10.1111/opn.70068

**Published:** 2026-03-08

**Authors:** Asa Inagaki‐Asano, Chie Fukui, Ayumi Igarashi, Mariko Sakka, Sameh Eltaybani, Maiko Noguchi‐Watanabe, Yoshinori Takeuchi, Noriko Yamamoto‐Mitani

**Affiliations:** ^1^ Graduate School of Medicine The University of Tokyo Tokyo Japan; ^2^ Global Nursing Research Centre, Graduate School of Medicine, The University of Tokyo Tokyo Japan; ^3^ Graduate School of Nursing Chiba University Chiba Japan; ^4^ Faculty of Medicine University of Tsukuba Tsukuba Japan; ^5^ Faculty of Medicine Institute of Science Tokyo Japan; ^6^ School of Data Science Yokohama City University Yokohama Japan

**Keywords:** aging, continuing care, family caregiving, home care, home care nursing, hospitalisation, longitudinal research, long‐term care, older people nursing, vulnerable adults

## Abstract

**Background:**

Preventing potentially avoidable hospitalisation (PAH) and reducing its duration are crucial to allow community‐dwelling older people to age at home. In Japan, homecare services, which include a variety of services such as home help, home‐based rehabilitation and home‐visit nursing, are covered by medical and long‐term care insurance and coordinated by care managers. Although home‐visit nursing is essential in homecare, studies investigating the association between the use of home‐visit nursing and the incidence and duration of PAH remain limited.

**Objective:**

To examine the association between home‐visit nursing use and the incidence and duration of PAH among community‐dwelling older people.

**Design:**

This was a 12‐month prospective cohort study.

**Study Settings:**

47 home‐visit nursing agencies and 73 care management offices across Japan.

**Participants:**

Older people (≥ 75 years) receiving homecare services.

**Methods:**

Using online questionnaires, home‐visit nurses and care managers reported older people's demographics, health status and PAH events over 12 months, as well as the state of using home‐visit nursing (users or non‐users). The incidence of PAH was dichotomised as either ‘none’ or ‘one and more’, due to heavy skewing. To examine PAH days, the rate of observed days was used due to the variability in the total observation period. Poisson regression and multivariate linear regression analyses were applied.

**Results:**

Of the 1450 participants initially recruited, 781 with complete dataset were included in the PAH incidence analysis. Of these, 81.0% were home‐visit nursing users. Mean participant age was 85.3 years (standard deviation: 6.1; range: 75–103), and 58.8% were female. The incidence rate ratio (IRR) of PAH was lower among home‐visit nursing users compared with non‐users (IRR, 0.63; 95% confidence interval [CI]: 0.41–0.95). Among 110 participants with PAH, there was no statistically significant difference in the rate of PAH days between home‐visit nursing users and non‐users (*β* = −0.65, 95% CI: −8.35–4.50).

**Conclusions:**

These results suggest that home‐visit nursing is associated with a lower incidence of PAH among older people; however, it is not associated with the duration of hospital stay once PAH occurs. For community‐dwelling older people with homecare services, home‐visit nursing may contribute to sustaining lives at home without PAH.

**Implications for Practice:**

Home‐visit nursing may help support community‐dwelling older people in remaining at home by minimizing the occurrence of PAH.

## Summary

**What does this research add to existing knowledge in gerontology?:**

- Home‐visit nursing use was associated with a lower incidence of PAH among community‐dwelling older people who need help from professional caregivers. There was no statistically significant difference in the duration of hospitalisation between home‐visit nursing users and non‐users.

**What are the implications of this new knowledge for nursing care with older people?:**

- It was suggested that home‐visit nursing use contributes to older people's longer, uninterrupted lives at home by preventing PAH.

**How Could the Findings be used to influence policy or practice or research or education?:**

- The findings can contribute to future intervention and database studies to explore the causal relationship between home‐visit nursing use and the incidence and duration of PAH. The findings indicated that home‐visit nursing service may be required to accommodate the increasing proportion of older people staying at home with minimum PAH.

## 1 Introduction

The increasingly aging population of developed countries poses significant demographic challenges. Integrated home‐based healthcare is a global movement that promotes healthy aging among older people (Rudnicka et al. 2020). Policymakers generally focus on the reconstruction of the long‐term care delivery system with integrated homecare to strike an appropriate balance between the total cost of medical and long‐term care insurance and demand from citizens (Worrall and Chaussalet 2015). In this context, potentially avoidable hospitalisation (PAH) is a valuable concept for evaluating the appropriateness of community or home‐based care.

PAH is defined as a hospitalisation event that could have been prevented if timely and appropriate homecare had been provided (Billings et al. 1993; Busby et al. 2017; Nyweide et al. 2013; Segal et al. 2014). In 1990, Solberg et al. identified 15 diagnoses (e.g., diabetes mellitus, asthma, congestive heart failure, pulmonary infection and cellulitis) that could be used to designate PAH through group discussions with several private insurance organisations in the United States (Solberg et al. 1990). PAH was subsequently used as a validated and useful indicator for the evaluation of home‐based healthcare quality in later studies (Billings et al. 1993; Caminal et al. 2004; Gao et al. 2014; Walsh et al. 2012; Weissman et al. 1992). Similar lists have been developed in other countries, such as the United Kingdom (Purdy et al. 2009), Australia (Ansari et al. 2002) and Canada (Ha et al. 2020; Haj‐Ali et al. 2020). One cross‐sectional study using 17 diagnoses previously identified in a study conducted in the United States (Walsh et al. 2012), found that they were also applicable in the Japanese context (Jeon et al. 2018). PAH can be considered a clinically important outcome indicator in Japan, as Japan is among the OECD countries with the highest number of hospital beds and the longest length of stay (OECD 2025). This context suggests that when a change in health status occurs at home, hospitalisation can be initiated relatively easily. Therefore, preventing PAH is a significantly meaningful outcome for older adults in community living.

Several studies have reported that homecare physicians contribute to the prevention of PAH. For instance, higher continuity of care (Chang et al. 2011; Godard‐Sebillotte et al. 2021), better access to homecare physicians (Kim et al. 2019) and higher numbers of homecare physicians and nurses per population (Daly et al. 2018; Mercier et al. 2020) were found to be associated with lower PAH incidence and length. Nurses are pivotal professionals who act as bridges between the medical and social care staff, working collaboratively with strong team dynamics (Jarrin et al. 2019). However, few studies have examined whether home‐visit nursing applications can contribute to the prevention of PAH or reduce its duration (Liu et al. 2020). Therefore, exploring the effectiveness of home‐visit nursing in preventing and reducing PAH is necessary.

In Japan, all citizens, including foreign residents, are covered by the National Health Insurance system, which includes medical and long‐term care services. The latter coverage was launched in 2000, and covers those aged 65 years and older and those aged 45 years and older with age‐related disabilities (i.e., designated incurable disease and end‐of‐life status) (Igarashi et al. 2020). Homecare services in Japan comprise a variety of services such as home‐visit nursing, home visits by physicians, home help and home‐based rehabilitation. The provision of homecare services, including home‐visit nursing, is covered by long‐term care insurance and coordinated by care managers in care management offices. In order to be classified as eligible for homecare services under long‐term care insurance, beneficiaries must undertake a nationally standardised examination which allows classification into one of seven care support/need levels: support care levels 1 and 2, and care need levels 1 through 5; people in care support level 1 are the most stable and independent, whereas those in care needs level 5 show the greatest deterioration and dependency (Igarashi et al. 2020). Based on beneficiaries' care support/need levels, home‐visit nursing may be prescribed as a homecare service. Home‐visit nursing is delivered by home‐visit nurses working at home‐visit nursing agencies and is available through medical and long‐term care insurance. Home‐visit nursing care includes, but is not limited to, physical assessment and symptom control, including patient education, caring for severe bedsores and control of medical equipment, such as mechanical ventilators. Previous research showed the effectiveness of home‐visit nursing to enable longer living at home (Oyama et al. 2013), clients' higher intentions to remain at home (Inagaki et al. 2021) and achievement of the desired home death (Ishikawa et al. 2017). However, to the best of our knowledge, no study has investigated the effectiveness of home‐visit nursing in preventing PAH or reducing its duration in Japan.

This study investigated the association between the use of home‐visit nursing services and incidence and duration of PAH. This study hypothesised that home‐visit nursing users would have a lower incidence and shorter duration of PAH than non‐users. We hope that the results of this investigation will provide valuable insights into the effectiveness of home‐visit nurses in promoting aging‐in‐place by avoiding PAH among community‐dwelling older people.

## 2 Materials and Methods

### 2.1 Study Design

This was a prospective cohort study conducted as a part of a larger project investigating the effectiveness of home‐visit nursing (Eltaybani et al. 2023). There are no duplicates or redundancies between the current study and other publications from the same research project. This study was reported in accordance with the STROBE (Strengthening the Reporting of Observational Studies in Epidemiology) guidelines (von Elm et al. 2007).

### 2.2 Study Settings

Home‐visit nursing agencies and care management offices were recruited at the national level through Japan's National Association for Visiting Nurse Services (https://www.zenhokan.or.jp/) and the Japan Care Manager Association (https://www.jcma.or.jp/), respectively. Before the study, home‐visit nursing agency managers who informally agreed to participate received an orientation from researchers on the detailed process (i.e., timing of follow‐up survey) and contents of the questionnaire in this study. A video explaining the survey process and instructions for completing the online survey, along with an accompanying guidebook, was sent to the care management offices' managers who had provided preliminary consent for the study. To minimise dropout and improve response rate, multiple reminders were sent to non‐respondents, and participants were frequently encouraged to contact the research team whenever difficulties or concerns regarding patient enrolment or data entry arose. As described above, care management offices act as the coordinators of homecare services, including home‐visit nursing provided under the long‐term care insurance system, while home‐visit nursing agencies are providers of home‐visit nursing only.

### 2.3 Participants

This study included older people receiving homecare services across Japan. The inclusion criteria for older people were: (1) age ≥ 75 years, (2) use of homecare services under long‐term care insurance and (3) having at least one of the following age‐related diagnoses: heart failure, chronic obstructive pulmonary disease, pneumonia, cerebrovascular disease, femoral neck fracture, cancer, nervous system disease, diabetes mellitus or dementia. Participants were classified into home‐visit nursing users and non‐users based on the type of homecare services received at baseline. Non‐users were defined as those who were using any kind of homecare services other than home‐visit nursing.

### 2.4 Sample Size

No formal a priori sample size calculations were performed. However, each home‐visit nursing agency and care management office were requested to recruit at least 25 and 5 older people, respectively. A sensitivity power analysis using the GPower software revealed that the current sample (*n* = 781) had a power of 90% to detect an effect size of W = 0.115 in *χ* ^2^ goodness‐of‐fit tests (two‐tailed alpha = 0.05, Degree of Freedom = 1) (Faul et al. 2007), which is the same effect size detected in a Korean study examining the differences in risk of PAH incidents according to the type of LTC insurance service in the elderly population (Kim and Lee 2020).

### 2.5 Data Collection

Data used for the current manuscript were collected from home‐visit nurses and care managers who were in charge of the targeted older people. Data were collected using online questionnaires on SurveyMonkey, and paper copies of the online questionnaire were available on request.

Data were collected at baseline and after 12 months at any point between September 2019 and May 2021. The observation period was defined as the time from the date of the initial survey response to the date of response to the follow‐up survey 12 months later. Multiple reminders were used to follow up on participants' responses. Respondents received pop‐up messages on their PCs when the response date was close. Email reminders were sent to agency managers in cases where they did not respond within a month.

### 2.6 Outcome Variables

PAH was defined as hospitalisation that may have been potentially preventable if healthcare professionals at home had provided care in a timely and appropriate manner. To identify PAH, we used a list of diagnoses developed in a previous Japanese study (Jeon et al. 2018). This list included 17 diagnoses: respiratory infection (acute bronchitis and pneumonia), congestive heart failure, urinary tract infection, weight loss, malnutrition, hypertension, falls or fractures (excluding fractures from motor vehicle accidents), diarrhoea, gastroenteritis, *Clostridium difficile* , chronic obstructive pulmonary disease, skin ulcers, cellulitis, altered mental status, acute confusion, delirium, electrolyte imbalance, constipation/fiscal impaction, seizures, sepsis, diabetes (poor glycaemic control) and anaemia. The respondents listed all admission and discharge dates during the observation period, which were calculated based on the response dates of the baseline and 12‐month surveys. In addition, they were asked to indicate whether each admission episode was a PAH.

The current study included two outcome variables: (i) the incidence of PAH and (ii) the number of PAH days during the observation period. All data of the current study, including the outcome variables, were reported by clinicians. In addition, they were asked to indicate whether each admission episode was a PAH. The incidence of PAH was calculated as the number of PAH detected during the observation period, referring to respondents' reported list of PAH. Older people were categorised into two groups: the PAH group included those who experienced at least one PAH, whereas the non‐PAH group included those who did not experience any PAH. This classification was applied because the data on the incidence of PAH were skewed. To examine the length of hospital stay due to PAH, we calculated the rate of hospital stay days out of the total observation period due to the variability in the overall observation period, which was introduced because the data were collected by busy practicing professionals when they could find the time.

### 2.7 Primary Independent Variable

Home‐visit nursing use was categorised as either ‘*yes*’ or ‘*no*’ based on the reported status at the baseline.

### 2.8 Covariates

**FIGURE 1:**
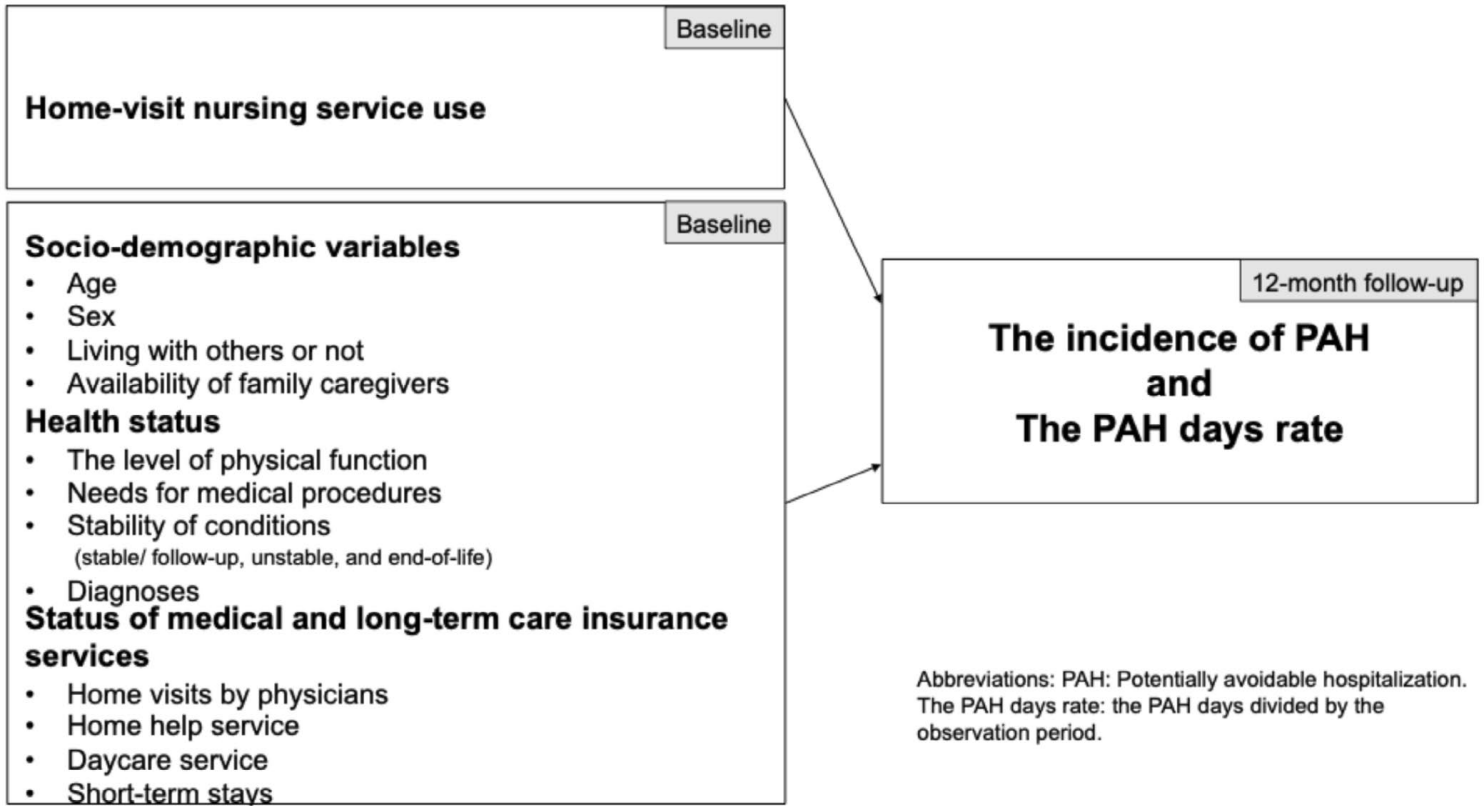
Conceptual framework.

All covariates were obtained at baseline and categorised into three domains: socio‐demographic variables, health status and the status of medical and long‐term care insurance services (Figure 1). Socio‐demographic variables included age, sex, living with others and availability of family caregivers. Health status included the level of physical function, whether medical procedures were needed, stability of the conditions and diagnoses. The level of physical function was reported using the levels designated by the Ministry of Health, Labour and Welfare (ranging from 1 to 4, with 4 indicating severe dependency) (Koyano et al. 1987). We defined 1–2 as ‘independent’ and 3–4 as ‘dependent’. Home‐visit nurses/care determined the older people's conditions as (1) stable/follow‐up, (2) unstable or (3) end‐of‐life. The diagnoses were classified as either present (1) or absent (0) for a range of diagnoses frequently observed in older people. (3) The status of medical and long‐term care insurance services was determined by whether older people used medical and long‐term care insurance services (i.e., home visits by physicians, home help service, daycare service and short‐stay services).

The duration of home‐visit nursing care use before the commencement of the study is a clinically important variable that may affect the outcomes of the current study. Therefore, we attempted to control for the potential confounding effects of this variable using two strategies. First, we prioritised participants with shorter durations of home‐visit nursing care. Second, we collected data on the duration of home‐visit nursing use before participating in the study. However, the latter variable (duration of home‐visit nursing use before participating in the study) had a large amount of missing data and was therefore not included in the current analysis.

### 2.9 Statistical Analysis

First, an attrition analysis was conducted to compare the characteristics of those who participated in the analysis and those who dropped out. Second, we examined the bivariate associations between independent variables at baseline and PAH incidence at 12‐month follow‐up. To compute the effect size in the bivariate analyses, we used the Phi‐coefficient (*ϕ*; for the chi‐square test and Fisher's exact test), Cohen's *d* (for Student's *t*‐test) and *r* (for Mann–Whitney's *U*‐test) (Wolverton et al. 2016). Third, after the bivariate analyses, we estimated the intention‐to‐treat incidence rate ratio (IRR) and 95% confidence interval (CI) for home‐visit nursing use by applying a Poisson regression model. An observational period‐weighted model with a robust variance structure was selected as the study period (365 days) differed among participants. This model was conditioned following covariates guided by previous studies: age (75–89, 90 ≤), sex (Magán et al. 2011), dependency of physical functions (dependent or independent), stability of their medical conditions (stable, unstable/terminal) (Gao et al. 2014), having secondary diagnosis (yes or no) (Muenchberger and Kendall 2010), and variables which were statistically significant (*p* < 0.05) in the bivariate analyses. To rule out the potential impact of care services other than home‐visit nursing, the multivariate analysis adjusted also for the use of various care services, namely home visit by physicians, short‐term stays, home help and day care service. Finally, multiple linear regression analysis was performed to explore the association between home‐visit nursing and the rate of PAH days among participants who experienced PAH during the study period (*n* = 110). The same confounders used in the analysis of PAH incidence were also included in this analysis. Analysis of the trajectory of participants in terms of using/non‐using home‐visit nursing revealed that a transition between the two statuses (i.e., use/non‐use) was found in less than 10% of participants. Therefore, an intention‐to‐treat analysis was conducted by classifying home‐visit nursing users and non‐users based on their baseline status.

IBM SPSS Statistics Premium Grand Pack ver. 29.0 for Mac OS was used for all the analyses. Statistical significance was set at *p*‐values less than 0.05.

### 2.10 Ethical Considerations

This investigation was conducted as part of a larger study and was approved by the Human Research Ethics Committee of the University of Tokyo (2019087NI‐(8)). The study purpose, the protection of information obtained from the completed questionnaire, the nature of voluntary participation and stating the completion and submission of the online questionnaire would be regarded as consent were explained to the study participants including home‐visit nurses, care managers, older people and their family members. Older people who participated in the study and their family members were all provided with an explanation of the study by their home‐visit nurses or care managers. Home‐visit nurses and care managers provided consent by checking a box before completing the web survey. Older people and family members obtained written consent and withdrew consent forms from home‐visit nurses or care managers.

## 3 Results

### 3.1 Study Sample

**FIGURE 2:**
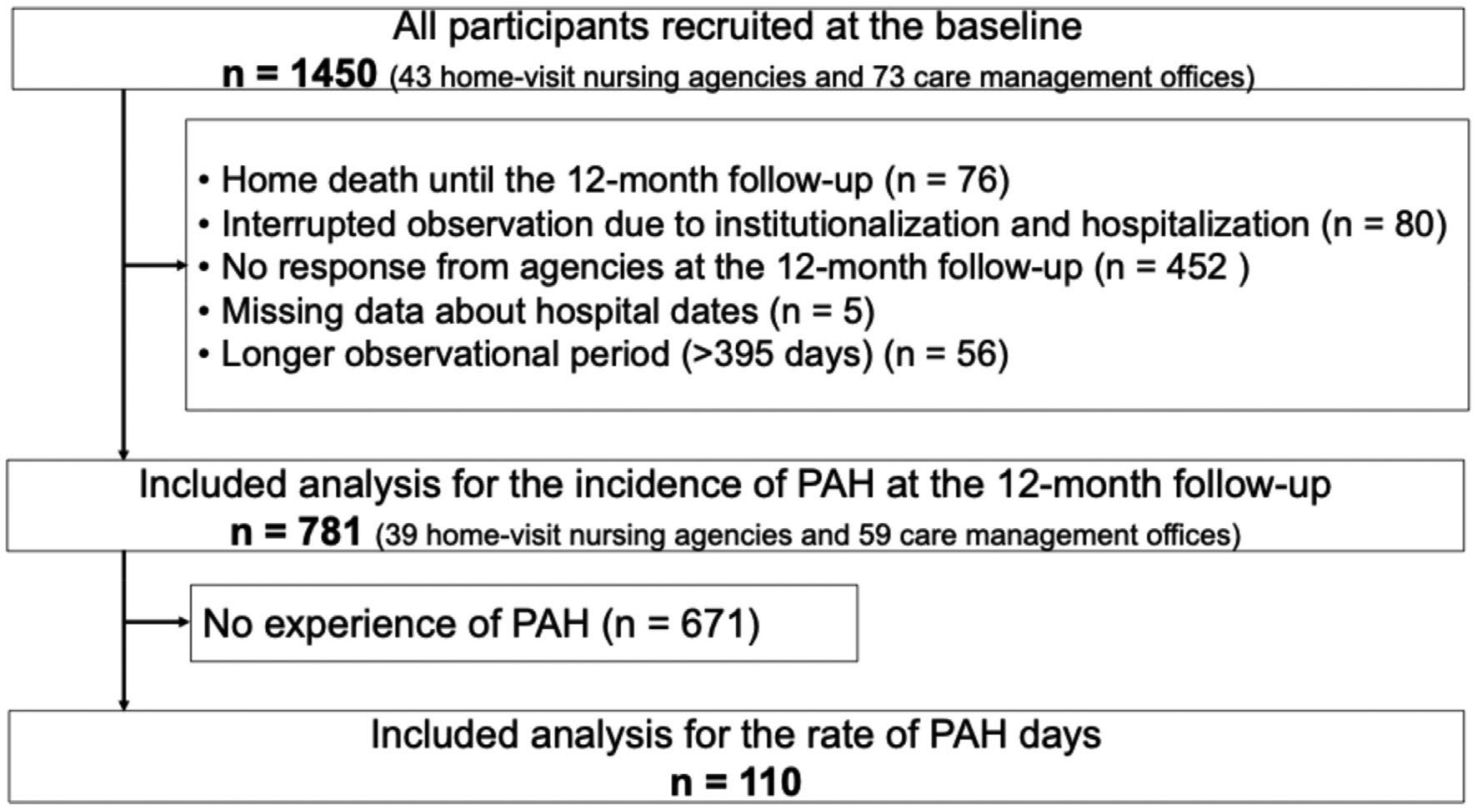
Participants flow.

At the baseline, we recruited 1450 older people from 47 home‐visit nursing agencies and 73 care management offices. Of these, 781 (53.9%) older people from 39 home‐visit nursing agencies and 59 care management offices had valid data from the 12‐month survey and were included in the analysis of the incidence of PAH. Reasons for exclusion include home death (*n* = 76), interrupted observation due to institutionalisation and hospitalisation (*n* = 80) and no response from agencies at 12‐month follow‐up (*n* = 452). Additionally, cases with missing data of hospital dates (*n* = 5) and longer observational periods (> 395 days) (*n* = 56) were excluded. After excluding participants without PAH (*n* = 671, 46.3%), 110 (7.6%) were included in the analysis of the PAH day rate (Figure 2). Table 1 shows that compared to those who were included in the analysis, those who dropped out included more home‐visit nursing users (*p* < 0.001; *ϕ* = 0.114), users of home visits by physicians (*p* < 0.001; *ϕ* = 0.103), patients at the end‐of‐life conditions (*p* < 0.001; *ϕ* = 0.147) and patients with cancer (*p* < 0.001; *ϕ* = 0.125).

**TABLE 1:** Attrition analysis comparing the characteristics of those who were included in the analysis and those who were excluded.

	Total (*n* = 1450)	Including analysis		*p*	Effect size^f^
		Yes (*n* = 781)	No (*n* = 669)		
**Primary independent variables**					
Home‐visit nursing use (Yes)	1230 (84.8)	633 (81.0)	597 (89.2)	< 0.001	0.114^c^
**Covariates**					
*Socio‐demographic variables*					
Age (Years)	85.3 ± 6.1	85.3 ± 6.13	84.8 ± 6.6	0.229	0.015^d^
90 years old ≤ (Yes)	416 (28.7)	169 (21.6)	247 (36.9)	0.555	0.015^c^
Gender (Female)	860 (59.3)	459 (58.8)	401 (59.9)	0.576	−0.015^c^
Living with others (No)	381 (26.3)	202 (25.9)	179 (26.8)	0.791	0.007^c^
Having a primary caregiver (Yes)	146 (10.1)	77 (9.9)	69 (10.3)	0.774	0.008^c^
*Health status*					
Level of physical functions^a^				0.137	0.039^c^
Independent	978 (67.4)	540 (69.1)	438 (65.5)		
Dependent	472 (32.6)	241 (30.9)	231 (34.5)		
Stability of conditions				< 0.001	0.147^c^
Stable	1130 (77.9)	638 (81.7)	492 (73.5)		
Unstable	247 (17.0)	127 (16.3)	120 (17.9)		
End‐of‐life	71 (4.9)	16 (2.0)	55 (8.2)		
Diagnosis					
Dementia	283 (19.5)	162 (20.7)	121 (18.1)	0.255	−0.030^c^
Cerebrovascular diseases	205 (14.1)	127 (16.3)	78 (11.7)	0.012	−0.066^c^
Heart failure	211 (14.6)	110 (14.1)	101 (15.1)	0.692	0.010^c^
Pulmonary infection	30 (2.1)	19 (2.4)	11 (1.6)	0.293	−0.028^e^
Cancer	222 (15.3)	87 (11.1)	135 (20.2)	< 0.001	0.125^c^
Diabetes mellitus	73 (5.0)	42 (5.4)	31 (4.6)	0.518	−0.017^c^
COPD	83 (5.7)	44 (5.6)	39 (5.8)	0.873	0.004^e^
Neurological disorder	108 (7.4)	56 (7.2)	52 (7.8)	0.663	0.011^c^
Gastro‐intestinal disease	20 (1.4)	14 (1.8)	6 (0.9)	0.145	−0.038^e^
Femoral neck fracture	44 (3.0)	27 (3.5)	17 (2.5)	0.311	−0.027^e^
Psychiatric disease	18 (1.2)	14 (1.8)	4 (0.6)	0.041	−0.056^e^
Others^b^	21 (1.4)	20 (2.6)	1 (0.1)	0.207	0.039^e^
Having secondary diagnoses (Yes)	1267 (87.4)	685 (87.7)	582 (87.0)	0.571	0.015^c^
Need for Medical procedures^g^	449 (31.0)	225 (28.8)	224 (33.5)	0.071	−0.047^c^
**Status of medical and long‐term care insurance services**					
*Medical insurance services* ^g^					
Non‐user of any medical insurance services	184 (12.7)	110 (14.1)	74 (11.1)	0.085	−0.045^c^
Outpatient	820 (56.6)	468 (59.9)	352 (52.6)	0.004	−0.076^c^
Home visits by physicians	521 (35.9)	245 (31.4)	276 (41.3)	< 0.001	0.103^c^
*Long‐term care insurance services* ^g^					
Home help	574 (39.6)	280 (35.9)	294 (43.9)	0.002	0.083^c^
Daycare	591 (40.8)	349 (44.7)	242 (36.2)	0.001	−0.086^c^
Short‐term stays	154 (10.6)	99 (12.7)	55 (8.2)	0.006	−0.072^c^

*Note:* Missing data were excluded from the analysis.

^a^ Ranged from 1 to 4; 4 means severe dependency. We dichotomised 1–2 as independent and 3–4 as dependent.

^b^ Including renal failure, musculoskeletal diseases, autoimmune diseases, cardiovascular diseases (except heart failure and cerebrovascular diseases) and osteoporosis.

^c^ Chi‐square test.

^d^ Student *t*‐test.

^e^ Fisher's exact test.

^f^ Cohen's *d* for Student‐test (0.2: weak, 0.5: moderate and 0.8: strong); *r* (calculated as z divided by the square root of N) for Mann–Whitney's *U*‐test; Phi‐coefficient for Chi‐square and Fisher's exact tests (0.1: weak, 0.3: moderate and 0.5: strong).

^g^ Multiple answers were allowed.

### 3.2 Participants' Characteristics

Tables 1, 2 present a descriptive analysis of the study participants. In this cohort, 633 participants (81.0%) used home‐visit nursing services. The mean age was 85.3 years (SD: 6.1, range: 75–103) and 169 (21.6%) were 90 years or older. There were 459 women (58.8%). Of the participants, 202 (25.9%) lived alone, 540 (69.1%) lived independently of physical dysfunction and 638 (81.7%) lived in a stable condition. Regarding the primary diagnosis, 162 (20.7%) participants had dementia, 127 (16.3%) had cerebrovascular disease, and 110 (14.1%) had heart failure. More than a one‐quarter of the participants did not require medical procedures (*n* = 225; 28.8%). Regarding the utilisation of medical and long‐term care services, 468 (59.9%) participants used outpatient medical care and 99 (12.7%) used short‐term stays.

**TABLE 2:** Participants' characteristics and association with the incidence of potentially avoidable hospitalisation.

	Potentially avoidable hospitalisation		*p*	Effect size^g^
	Yes (*n* = 110)	No (*n* = 671)		
**Primary independent variable**				
Home‐visit nursing use (Yes)	83 (75.5)	550 (82.0)	0.106	−0.060^c^
**Outcome variables (Potentially Avoidable Hospitalisation; Yes)**				
Total days	51 ± 46.2	—^d^		
0		671 (100.0)		
1–50	68 (61.8)			
51–100	24 (21.8)	—		
101–150	12 (10.9)	—		
151–200	5 (4.5)	—		
201–250	1 (0.9)	—		
Days rate^h^	13.9 ± 12.8	—^e^		
**Covariates**				
*Socio‐demographic variables*				
Age (Years)	85.5 ± 6.0	85.2 ± 6.2	0.558	0.016^d^
90 years old ≤	23 (20.9)	146 (21.8)	0.841	0.007^c^
Gender (female)	68 (61.8)	391 (58.3)	0.484	0.025^c^
Living with others (No)	17 (15.5)	185 (27.6)	0.007	−0.096^c^
Having a primary caregiver (Yes)	102 (92.7)	602 (89.7)	0.326	0.035^c^
**Health status**				
*Level of physical functions* ^a^				
Independent	78 (70.9)	548 (81.7)	0.072	0.056
Dependent	46 (41.8)	225 (33.5)		
*Stability of conditions*				
Stable	87 (79.1)	551 (82.1)	0.681	0.031^c^
Unstable	21 (19.1)	106 (15.8)		
End‐of‐life	2 (1.8)	14 (2.1)		
Diagnosis				
Dementia	14 (12.7)	148 (22.1)	0.025	−0.080^c^
Cerebrovascular diseases	21 (19.1)	106 (15.8)	0.386	0.031^c^
Heart failure	22 (20.0)	88 (13.1)	0.054	0.069^c^
Pulmonary infection	7 (6.4)	12 (1.8)	0.004	0.103^c^
Cancer	8 (7.3)	79 (11.8)	0.164	0.050^c^
Diabetes mellitus	8 (7.3)	34 (5.1)	0.342	0.034^c^
COPD	9 (8.2)	35 (5.2)	0.211	0.045^c^
Neurological disorder	8 (7.3)	48 (7.2)	0.964	0.027^c^
Gastro‐intestinal disease	1 (0.9)	13 (1.9)	0.451	0.056^f^
Femoral neck fracture	1 (0.9)	26 (3.9)	0.115	0.027^f^
Psychiatric disease	1 (0.9)	13 (1.9)	0.451	0.019^f^
Others^b^	2 (1.8)	18 (2.7)	0.595	0.034^f^
Having secondary diagnoses (Yes)	104 (94.5)	581 (86.6)	0.018	0.084^c^
Need for Medical procedures^i^	45 (40.9)	180 (26.8)	0.003	0.108^c^
**Status of medical and long‐term care insurance services**				
*Medical insurance services* ^i^				
Non‐user of any medical insurance services	21 (19.1)	98 (14.6)	0.194	0.045^c^
Outpatient	67 (60.9)	401 (59.8)	0.820	0.008^c^
Home visits by physicians	28 (25.5)	217 (32.3)	0.149	0.052^c^
*Long‐term care insurance services* ^i^				
Home help	31 (28.2)	249 (37.1)	0.070	0.065^c^
Daycare	15 (13.6)	86 (12.8)	0.812	0.008^c^
Short‐term stays	23 (20.9)	76 (11.3)	0.005	0.100^c^

*Note:* Missing data were excluded from the analysis.

^a^ Ranged from 1 to 4; 4 means severe dependency. We dichotomised 1–2 as independent and 3–4 as dependent.

^b^ Including renal failure, musculoskeletal diseases, autoimmune diseases, cardiovascular diseases (except heart failure and cerebrovascular diseases) and osteoporosis.

^c^ Chi‐square test.

^d^ Student *t*‐test.

^e^ Mann–Whitney's *U*‐test.

^f^ Fisher's exact test.

^g^ Cohen's *d* for Student‐test (0.2: weak, 0.5: moderate and 0.8: strong); *r* (calculated as *z* divided by the square root of *N*) for Mann Whitney's *U*‐test; Phi‐coefficient for Chi‐square and Fisher's exact tests (0.1: weak, 0.3: moderate and 0.5: strong).

^h^ Computed by dividing the total PAH days by each participant's observational period.

^i^ Multiple answers were required.

### 3.3 PAH and PAH Days

Overall, 110 participants (14.1%) experienced PAH during the study period. The total PAH days ranged from 0 to 203, and most participants (671, 85.9%) had no history of PAH. The details are presented in Table 2.

### 3.4 Factors Associated With the Incidence of PAH (Bivariate Analysis)

Table 2 shows the bivariate associations between the participants' characteristics and incidence of PAH. Living alone (*p* = 0.007; *ϕ* = −0.096) and dementia as a primary diagnosis (*p* = 0.025; *ϕ* = −0.080) were both negatively associated with the incidence of PAH. In contrast, pulmonary infection (*p* = 0.004; *ϕ* = 0.103), having secondary diagnoses (*p* = 0.018; *ϕ* = 0.084), needing medical procedures (*p* = 0.003; *ϕ* = 0.108) and use of short‐stay services as the long‐term care insurance services (*p* = 0.005; *ϕ* = 0.100) were positively associated with the incidence of PAH.

### 3.5 Association Between Home‐Visit Nursing Use and the Incidence of PAH and the PAH Days Rate

The associations between home‐visit nursing use and the incidence of PAH were examined in all participants (*n* = 781), while the association between home‐visit nursing use and PAH day rate was examined only in participants with PAH (*n* = 110). The bivariate analysis (Table 3) showed that home‐visit nursing use was not statistically significant with either the incidence of PAH (*p* = 0.106; *ϕ* = −0.060) or PAH day rate (*p* = 0.278; *r* = −0.203). The multivariate Poisson regression model (Table 4) revealed that home‐visit nursing users had a statistically significant lower incidence of PAH (Incidence Rate Ratio [IRR]: 0.63, 95% confidence interval [95% CI]: 0.41–0.95). The multivariate linear regression model (Table 4) showed no statistically significant differences in the rate of PAH days between home‐visit nursing users and non‐users (coefficient [*β*]: −0.65, 95% CI: −8.35–4.50).

**TABLE 3:** Bivariate association between home‐visit nursing use and the incidence and duration of potentially avoidable hospitalisation.

**Home‐visit nursing use**	**Potentially avoidable hospitalisation (*N* = 781)**			
	Yes (*n* = 110) *n* (%)		No (*n* = 671) *n* (%)	*p* ^a^ (Effect size)
Yes	83 (13.1)		550 (86.9)	0.106
No	27 (18.2)		121 (81.8)	(−0.06)
	**Rate of potentially avoidable hospitalisation days (*N* = 110)** ^c^			
	Mean	SD	[range]	*p* ^b^ (Effect size)
Yes	13.2	±11.9	[0.3–53.5]	0.278
No	16.3	±15.2	[0.5–55.3]	(−0.203)

Abbreviations: SD; standard deviation.

^a^ *χ* ^2^‐test with Phi‐coefficient as an effect size (0.1: weak, 0.3: moderate and 0.5: strong).

^b^ Mann–Whitney's *U*‐test with *r* (calculated as *z* divided by the square root of *N*) as an effect size.

^c^ Participants with the incidence of potentially avoidable hospitalisation.

**TABLE 4:** Multivariate association between home‐visit nursing use and the incidence and duration of potentially avoidable hospitalisation.

**Potentially avoidable hospitalisation (*N* = 781)**	
IRR^a^	[95% CI]
0.63	[0.41–0.95]
**Potentially avoidable hospitalisation days rate (*N* = 110)**	
*β* ^b^	[95% CI]
−0.65	[−8.35 to 4.50]

*Note:* Shown are the regression coefficients of the association between home‐visit nursing use adjusted confounders: (1) socio‐demographics; age 90 ≤, gender, living alone, (2) Health status; Level of physical functions, stability of conditions, diagnosis (dementia, heart failure and pulmonary infection), having secondary diagnosis, needing for medical procedures, (3) Status of medical and long‐term care insurance services; Home visit by physicians, Short‐term stays, Home help, Day care service.

Abbreviations: CI, confidence interval; IRR, incidence rate ratio.

^a^ A Poisson regression model.

^b^ A multivariate regression analysis.

## 4 Discussion

The current study examined the association between home‐visit nursing use and the incidence of PAH and the PAH days rate among community‐dwelling older people. Overall, the results showed that home‐visit nursing users had a lower incidence of PAH than non‐users during the observation period. However, there were no statistically significant differences in the PAH days rate between home‐visit nursing users and non‐users. To the best of our knowledge, this is among the first studies to examine the association between home‐visit nursing and the incidence and duration of PAH. Several systematic reviews have previously reported controversial results regarding the effectiveness of home‐visit nursing in reducing the incidence and length of hospitalisation (Laurant et al. 2018; Mayo‐Wilson et al. 2014). As such, our findings offer new insights into the association between home‐visit nursing use in Japan and expanded intervals between PAHs, which might enable more ‘Aging in Place’ (Owusu et al. 2023).

The cumulative number of home‐based long‐term care service users in Japan reached 50.59 million person‐visits per year by 2025. Under Japan's public long‐term care insurance system, approximately 70% of service users are classified as mild functional dependency (Ministry of Health, Labour and Welfare 2025). In our study sample, 69.1% of participants were physically independent, indicating that their functional status is broadly comparable to that of home‐based service users nationwide. Regarding primary disease types, among individuals aged 75 years and older nationwide, the leading causes of home‐based care are dementia (17.9%), cerebrovascular diseases (12.8%), cardiovascular diseases including heart failure (5.5%) and cancer (2.5%) (Ministry of Health, Labour and Welfare 2022). In the present study, the prevalence of these diagnoses was higher than the national estimates. This finding suggests that, relative to the broader population of care‐dependent older people aged 75 and over, the participants in this study had more frequent conditions requiring ongoing management, including symptom control.

In the present study, the number of patients lost at the follow‐up was relatively high. One major reason for the high dropout is the COVID‐19 pandemic. The high attrition rate is a documented problem in many studies conducted amid the pandemic in Japan (Shinohara et al. 2021) and other countries (Xiang et al. 2024; da Graca et al. 2022). Like many other countries (Allan et al. 2021; Markkanen et al. 2021), many homecare agencies in Japan were extensively involved in emergency procedures related to COVID‐19, negatively affecting their capacity to participate in research activities. This explains why the largest percentage of those who dropped out in the current study were due to unavailability of the care management offices and home‐visit nursing agencies.

Another possible explanation for the high dropout in the current study is the poor health status of the study participants. This is evidenced by the findings that the use of home‐visit nursing and home‐visit physicians, as well as those with cancer and end‐of‐life conditions, was higher among those who dropped out. This demonstrates the challenges of prospective data collection from older people, especially those with health status requiring support. Future prospective research needs to balance the benefits of long follow‐up intervals and the potential for high dropout. Furthermore, the development and use of integrated datasets spanning long‐term care facilities, hospitals and home care settings would be essential for prospectively and continuously tracking the health trajectories of older people who typically move across various care settings and might die at different places from home.

A favourable association was observed between home‐visit nursing and the incidence of PAH. Three mechanisms might have contributed to this result. First, home‐visit nurses might manage older people's symptoms and disease exacerbations, leading to a lower incidence of PAH. Nurse‐led home‐visiting programs have been shown to reduce the incidence of chronic obstructive pulmonary disease exacerbations (Ram et al. 2004) and emergency department visits due to disease exacerbation (Osakwe et al. 2020). Second, home‐visit nurses may empower older people to enhance their self‐care management, which helps prevent disease worsening and thereby reduces the incidence of PAH. Previous studies revealed that nurse‐led educational home interventions can improve client self‐management and prevent unfavourable health outcomes (Cho and Kim 2021). Third, home‐visit nursing may help lower family caregiver burden and increase their satisfaction (Stojak et al. 2019). Previous research found that a higher caregiver burden leads to more client hospitalisations (Kuzuya et al. 2011).

The current study found no significant association between home‐visit nursing care and hospitalisation duration. Furthermore, the effect of home‐visit nursing on reducing the number of hospitalisation days has not been clearly demonstrated in existing systematic reviews and meta‐analyses. Although it has been reported that family caregivers with home‐visit nursing have a reduced sense of burden at the time of discharge (Harada et al. 2013) and that home‐visit nursing facilitates the smooth transfer of medical and nursing information (Kawashima et al. 2017), its effect on reducing hospitalisation duration has not been confirmed. Hospitals with discharge support departments and a small number of beds have been associated with shorter average hospitalisation days (Busby et al. 2017; Provencher et al. 2020). This study did not adjust for such covariates, which may have influenced the result.

Another possible reason for the lack of significance in terms of hospitalisation duration is the small sample size. In this study, the analysed subjects included only those who had experienced PAH at least once during the observation period, which might have limited the statistical power. Therefore, further studies with larger sample sizes are warranted. Furthermore, this limitation may be due to differences in the basic characteristics of home care users and non‐users. Overall, we found that home‐visit nursing users were more vulnerable, which may have affected the results even though we attempted to control for these variables using multivariate regression analyses. Among older people, a poorer general condition is associated with a prolonged hospital stay (Lin et al. 2016), as well as a lower likelihood of discharge to home (Fortinsky et al. 1999). Therefore, the lack of a significant difference in hospitalisation days suggests the effectiveness of home‐visit nursing for patients with extensive medical conditions.

### 4.1 Clinical Implications

The current study showed that home‐visit nursing users had a lower incidence of PAH than non‐users, although there were no statistically significant differences in hospitalisation days among the participants who experienced PAH. Despite cross‐national variability in PAH‐related conditions, the list of diseases used in the current study has been shown to be valid in the Japanese context, while remaining consistent with international standards. Therefore, the current results provide insights that are not only contextually relevant to Japan but also relevant to other healthcare contexts. The results suggest that home‐visit nurses may contribute to older people's longer, uninterrupted lives at home by preventing PAH that can cause serious deterioration in frail older people. Future intervention and community‐based representative database studies need to confirm the current results by exploring the causal relationship between home‐visit nursing use and the incidence and duration of PAH. Policymakers and clinicians need to consider promoting the use of home‐visit nursing among frail older people living in the community to maintain continuity of care and prevent disruptions to home‐based living.

## 5 Limitations

Certain limitations of the current study merit mention. First, the generalisability of the results is hindered by the fact that data were only available for participants who remained in the study for the full 12‐month follow‐up period. In addition, although the study targeted home‐visit nursing agencies and care management agencies nationwide, random sampling or other probability‐based sampling methods were not employed. Therefore, the national representativeness of the finding is limited. Hospitalisation‐related data of those who dropped out could not be obtained. This is particularly important given the results of the attrition analysis that showed that those who dropped out were more vulnerable and at end‐of‐life stage conditions compared to those who were included in the analysis. Moreover, the study population consisted of individuals with more severe disease profiles than the national average, which may have further limited the ability to detect significant associations. Together, these factors may have introduced selection bias and restricted the applicability of an intent‐to‐treat analysis. Future research should explore strategies to reduce attrition and consider alternative analytic approaches to account for missing data.

Second, the analysis could not include certain factors that might have affected PAH or its duration, such as the length of HVN use and the density of hospitals or homecare clinics in the community. It is noteworthy that Japan has adopted a national health insurance system characterised by the availability of fairly standardised medical and nursing care services, and therefore, the possible effect of community characteristics may be minimal.

Third, the data validity was limited, as the coding of the incidence of PAH and its duration was not verified. Therefore, their accuracy depends on the discretion of the respondents. In Japan, client data recorded by homecare service providers is processed independently by each office without integration. Therefore, despite the limitation imposed by the self‐reporting of the study outcome, no other feasible, more valid or reliable approach might be available. Therefore, future development of an integrated database system would help validate such key data. Finally, the incidence of PAH must be higher in patients with higher care needs. In the future, stratified analysis and risk adjustment by level of care requirement will be considered.

## 6 Conclusions

The present study investigated the effectiveness of home‐visit nursing on reducing the incidence and duration of PAH using data from a longitudinal prospective cohort of community‐dwelling older people aged 75 years and older. We found that home‐visit nursing users had a 40% lower incidence of PAH than non‐users, although there was no significant difference in the total number of PAH hospitalisation days. These results indicate that home‐visit nursing use may contribute to longer, uninterrupted living at home among older people.

## Author Contributions

**Asa Inagaki‐Asano:** conceptualisation, methodology, formal analysis, data curation, writing – original draft, writing – review and editing and visualisation. **Chie Fukui:** conceptualisation, methodology, data curation, writing – review and editing. **Ayumi Igarashi:** conceptualisation, methodology, writing – review and editing. **Mariko Sakka:** conceptualisation, methodology, writing – review and editing. **Sameh Eltaybani:** formal analysis, writing – review and editing. **Maiko Noguchi‐Watanabe:** conceptualisation, methodology, writing – review and editing. **Yoshinori Takeuchi:** methodology, formal analysis, writing – review and editing. **Noriko Yamamoto‐Mitani:** project administration, funding acquisition, supervision, conceptualisation, methodology, writing – review and editing.

## Funding

Funding for this study was supported by a Grant for Geriatric Health Promotion Project provided by the Japanese Ministry of Health, Labor, and Welfare (number: 21GA1002; grant provided to Noriko Yamamoto‐Mitani).

## Conflicts of Interest

The authors declare no conflicts of interest.

## Data Availability

The data that support the findings of this study are available on request from the corresponding author. The data are not publicly available due to privacy or ethical restrictions.

## References

[opn70068-bib-0001] Allan, P. J. , L. Pironi , F. Joly , et al. 2021. “An International Survey of Clinicians' Experience Caring for Patients Receiving Home Parenteral Nutrition for Chronic Intestinal Failure During the COVID‐19 Pandemic.” JPEN Journal of Parenteral and Enteral Nutrition 45, no. 1: 43–49. 10.1002/jpen.2050. 33241555 PMC7753815

[opn70068-bib-0002] Ansari, Z. , N. Carson , A. Serraglio , T. Barbetti , and F. Cicuttini . 2002. “The Victorian Ambulatory Care Sensitive Conditions Study: Reducing Demand on Hospital Services in Victoria.” Australian Health Review 25: 71–77. 10.1071/ah020071. 12046157

[opn70068-bib-0003] Billings, J. , L. Zeitel , J. Lukomnik , T. S. Carey , A. E. Blank , and L. Newman . 1993. “Impact of Socioeconomic Status on Hospital Use in New York City.” Health Affairs 12, no. 1: 162–173. 10.1377/hlthaff.12.1.162. 8509018

[opn70068-bib-0004] Busby, J. , S. Purdy , and W. Hollingworth . 2017. “How Do Population, General Practice and Hospital Factors Influence Ambulatory Care Sensitive Admissions: A Cross Sectional Study.” BMC Family Practice 18, no. 1: 67. 10.1186/s12875-017-0638-9. 28545412 PMC5445441

[opn70068-bib-0005] Caminal, J. , B. Starfield , E. Sánchez , C. Casanova , and M. Morales . 2004. “The Role of Primary Care in Preventing Ambulatory Care Sensitive Conditions.” European Journal of Public Health 14, no. 3: 246–251. 10.1093/eurpub/14.3.246. 15369028

[opn70068-bib-0006] Chang, C. H. , T. A. Stukel , A. B. Flood , and D. C. Goodman . 2011. “Primary Care Physician Workforce and Medicare Beneficiaries' Health Outcomes.” JAMA 305, no. 20: 2096–2104. 10.1001/jama.2011.665. 21610242 PMC3108147

[opn70068-bib-0007] Cho, M.‐K. , and M. Y. Kim . 2021. “Self‐Management Nursing Intervention for Controlling Glucose Among Diabetes: A Systematic Review and Meta‐Analysis.” International Journal of Environmental Research and Public Health 18, no. 23: 12750. 10.3390/ijerph182312750. 34886488 PMC8657503

[opn70068-bib-0008] da Graca, B. , L. R. Hall , K. Sanchez , M. M. Bennett , M. B. Powers , and A. M. Warren . 2022. “The Risks of Attrition Bias in Longitudinal Surveys of the Impact of COVID‐19.” Proceedings 36, no. 2: 161–164. 10.1080/08998280.2022.2139541. PMC9980691 36876266

[opn70068-bib-0009] Daly, M. R. , J. M. Mellor , and M. Millones . 2018. “Do Avoidable Hospitalization Rates Among Older Adults Differ by Geographic Access to Primary Care Physicians?” Health Services Research 53, no. Suppl 1: 3245–3264. 10.1111/1475-6773.12736. 28660679 PMC6056577

[opn70068-bib-0010] Eltaybani, S. , S. Kitamura , C. Fukui , et al. 2023. “Toward Developing Care Outcome Quality Indicators for Homecare for Older People: A Prospective Cohort Study in Japan.” Geriatrics & Gerontology International 23, no. 5: 383–394. 10.1111/ggi.14578. 37132041 PMC11503618

[opn70068-bib-0011] Faul, F. , E. Erdfelder , A.‐G. Lang , and A. Buchner . 2007. “G*Power 3: A Flexible Statistical Power Analysis Program for the Social, Behavioral, and Biomedical Sciences.” Behavior Research Methods 39: 175–191. 10.3758/BF03193146. 17695343

[opn70068-bib-0012] Fortinsky, R. , K. Covinsky , R. Palmer , and C. Landefeld . 1999. “Effects of Functional Status Changes Before and During Hospitalization on Nursing Home Admission of Older Adults.” Journals of Gerontology. Series A, Biological Sciences and Medical Sciences 54: M521–M526. 10.1093/gerona/54.10.m521. 10568535

[opn70068-bib-0013] Gao, J. , E. Moran , Y.‐F. Li , and P. L. Almenoff . 2014. “Predicting Potentially Avoidable Hospitalizations.” Medical Care 52, no. 2: 164–171. 10.1097/MLR.0000000000000041. 24374413

[opn70068-bib-0014] Godard‐Sebillotte, C. , E. Strumpf , N. Sourial , L. Rochette , E. Pelletier , and I. Vedel . 2021. “Primary Care Continuity and Potentially Avoidable Hospitalization in Persons With Dementia.” Journal of the American Geriatrics Society 69, no. 5: 1208–1220. 10.1111/jgs.17049. 33635538

[opn70068-bib-0015] Ha, N. T. , C. Wright , D. Youens , D. B. Preen , and R. Moorin . 2020. “Effect Modification of Multimorbidity on the Association Between Regularity of General Practitioner Contacts and Potentially Avoidable Hospitalisations.” Journal of General Internal Medicine 35, no. 5: 1504–1515. 10.1007/s11606-020-05699-0. 32096082 PMC7210343

[opn70068-bib-0016] Haj‐Ali, W. , R. Moineddin , B. Hutchison , W. P. Wodchis , and R. H. Glazier . 2020. “Role of Interprofessional Primary Care Teams in Preventing Avoidable Hospitalizations and Hospital Readmissions in Ontario, Canada: A Retrospective Cohort Study.” BMC Health Services Research 20, no. 1: 782. 10.1186/s12913-020-05658-9. 32831072 PMC7444082

[opn70068-bib-0017] Harada, S. , M. Sugimoto , M. Akiyama , T. Okada , and S. Sakurai . 2013. “Analysis of Participation in Discharge Planning by Visiting Nurse: From a Comparison After Typical Discharge Planning by a Hospital.” Juntendo Medical Journal 59: 480–489. 10.14789/jmj.59.480.

[opn70068-bib-0018] Igarashi, A. , S. Eltaybani , M. Takaoka , M. Noguchi‐Watanabe , and N. Yamamoto‐Mitani . 2020. “Quality Assurance in Long‐Term Care and Development of Quality Indicators in Japan.” Gerontology & Geriatric Medicine 6: 2333721420975320. 10.1177/2333721420975320. 35047653 PMC8762483

[opn70068-bib-0019] Inagaki, A. , M. Noguchi‐Watanabe , M. Sakka , and N. Yamamoto‐Mitani . 2021. “Home‐Visit Nurses' Community Involvement Activities and Preference Regarding the Place for End‐Of‐Life Period Among Single Older Adults: A Cross‐Sectional Study in Japan.” Health & Social Care in the Community 29, no. 5: 1584–1593. 10.1111/hsc.13224. 33211365

[opn70068-bib-0020] Ishikawa, T. , S. Fukui , and Y. Okamoto . 2017. “Association Between Advance Care Planning by a Visiting Nurse and Achieving the Desired Place of Death for Patients With End‐Stage Cancer.” Japan Journal of Nursing Science 37: 123–131. 10.5630/jans.37.123.

[opn70068-bib-0021] Jarrin, O. F. , F. A. Pouladi , and E. A. Madigan . 2019. “International Priorities for Home Care Education, Research, Practice, and Management: Qualitative Content Analysis.” Nurse Education Today 73: 83–87. 10.1016/j.nedt.2018.11.020. 30550942 PMC6713276

[opn70068-bib-0022] Jeon, B. , N. Tamiya , S. Yoshie , K. Iijima , and T. Ishizaki . 2018. “Potentially Avoidable Hospitalizations, Non‐Potentially Avoidable Hospitalizations and In‐Hospital Deaths Among Residents of Long‐Term Care Facilities.” Geriatrics & Gerontology International 18, no. 8: 1272–1279. 10.1111/ggi.13458. 30136395

[opn70068-bib-0023] Kawashima, M. , M. Mori , and A. Isobe . 2017. “The Actual State of the Information Gathering on Home Care Shift Patients by Visiting Nurses Before the First Visit.” Seisen Journal of Nursing Studies 6: 75–82.

[opn70068-bib-0024] Kim, A. M. , J. H. Park , T. H. Yoon , and Y. Kim . 2019. “Hospitalizations for Ambulatory Care Sensitive Conditions as an Indicator of Access to Primary Care and Excess of Bed Supply.” BMC Health Services Research 19, no. 1: 259. 10.1186/s12913-019-4098-x. 31029134 PMC6487016

[opn70068-bib-0025] Kim, J. H. , and Y. Lee . 2020. “Potentially Avoidable Hospitalization Among Long‐Term Care Insurance Beneficiaries With Dementia.” Korean Journal of Family Medicine 41, no. 5: 318–324. 10.4082/kjfm.18.0184. 32316707 PMC7509129

[opn70068-bib-0026] Koyano, W. , H. Shibata , H. Haga , Y. Suyama , and K. Nakazato . 1987. “Measurment of Competence in the Elderly Living at Home Development of an Index of Competence.” Japanese Journal of Public Health 3: 109–114.

[opn70068-bib-0027] Kuzuya, M. , H. Enoki , J. Hasegawa , et al. 2011. “Impact of Caregiver Burden on Adverse Health Outcomes in Community‐Dwelling Dependent Older Care Recipients.” American Journal of Geriatric Psychiatry 19, no. 4: 382–391. 10.1097/JGP.0b013e3181e9b98d. 20808120

[opn70068-bib-0028] Laurant, M. , M. van der Biezen , N. Wijers , K. Watananirun , E. Kontopantelis , and A. J. van Vught . 2018. “Nurses as Substitutes for Doctors in Primary Care.” Cochrane Database of Systematic Reviews 7, no. 7: CD001271. 10.1002/14651858.CD001271.pub3. 30011347 PMC6367893

[opn70068-bib-0029] Lin, H. S. , J. N. Watts , N. M. Peel , and R. E. Hubbard . 2016. “Frailty and Post‐Operative Outcomes in Older Surgical Patients: A Systematic Review.” BMC Geriatrics 16, no. 1: 157. 10.1186/s12877-016-0329-8. 27580947 PMC5007853

[opn70068-bib-0030] Liu, C. F. , P. L. Hebert , J. H. Douglas , et al. 2020. “Outcomes of Primary Care Delivery by Nurse Practitioners: Utilization, Cost, and Quality of Care.” Health Services Research 55, no. 2: 178–189. 10.1111/1475-6773.13246. 31943190 PMC7080399

[opn70068-bib-0031] Magán, P. , Á. Alberquilla , Á. Otero , and J. M. Ribera . 2011. “Hospitalizations for Ambulatory Care Sensitive Conditions and Quality of Primary Care: Their Relation With Socioeconomic and Health Care Variables in the Madrid Regional Health Service (Spain).” Medical Care 49, no. 1: 17–23. http://www.jstor.org/stable/25767031. 20978453 10.1097/MLR.0b013e3181ef9d13

[opn70068-bib-0032] Markkanen, P. , N. Brouillette , M. Quinn , et al. 2021. ““It Changed Everything”: The Safe Home Care Qualitative Study of the COVID‐19 Pandemic's Impact on Home Care Aides, Clients, and Managers.” BMC Health Services Research 21, no. 1: 1055. 10.1186/s12913-021-07076-x. 34610836 PMC8491760

[opn70068-bib-0033] Mayo‐Wilson, E. , S. Grant , J. Burton , A. Parsons , K. Underhill , and P. Montgomery . 2014. “Preventive Home Visits for Mortality, Morbidity, and Institutionalization in Older Adults: A Systematic Review and Meta‐Analysis.” PLoS One 9, no. 3: e89257. 10.1371/journal.pone.0089257. 24622676 PMC3951196

[opn70068-bib-0034] Mercier, G. , V. Georgescu , E. Plancque , C. Duflos , A. Le Pape , and C. Quantin . 2020. “The Effect of Primary Care on Potentially Avoidable Hospitalizations in France: A Cross‐Sectional Study.” BMC Health Services Research 20, no. 1: 268. 10.1186/s12913-020-05132-6. 32234078 PMC7106616

[opn70068-bib-0035] Ministry of Health, Labour and Welfare . 2022. “National Health and Nutrition Survey 2022: Overview [PDF].” (In Japanese). https://www.e‐stat.go.jp/dbview?sid=0002041125&utm_source.com.

[opn70068-bib-0036] Ministry of Health, Labour and Welfare . 2025. “Overview of the FY2023 Long‐Term Care Insurance Business Report [PDF].” (In Japanese). https://www.mhlw.go.jp/topics/kaigo/osirase/jigyo/23/dl/r05_gaiyou.pdf.

[opn70068-bib-0038] Muenchberger, H. , and E. Kendall . 2010. “Predictors of Preventable Hospitalization in Chronic Disease: Priorities for Change.” Journal of Public Health Policy 31, no. 2: 150–163. 10.1057/jphp.2010.3. 20535098

[opn70068-bib-0039] Nyweide, D. J. , D. L. Anthony , J. P. Bynum , et al. 2013. “Continuity of Care and the Risk of Preventable Hospitalization in Older Adults.” JAMA Internal Medicine 173, no. 20: 1879–1885. 10.1001/jamainternmed.2013.10059. 24043127 PMC3877937

[opn70068-bib-0040] OECD . 2025. Health at a Glance 2025. OECD Publishing. 10.1787/8f9e3f98-en.

[opn70068-bib-0041] Osakwe, Z. T. , S. Aliyu , O. A. Sosina , and L. Poghosyan . 2020. “The Outcomes of Nurse Practitioner (NP)‐Provided Home Visits: A Systematic Review.” Geriatric Nursing 41, no. 6: 962–969. 10.1016/j.gerinurse.2020.07.001. 32718756 PMC7380935

[opn70068-bib-0042] Owusu, B. , B. Bivins , B. R. Marseille , and D. L. Baptiste . 2023. “Aging in Place: Programs, Challenges and Opportunities for Promoting Healthy Aging for Older Adults.” Nursing Open 10, no. 9: 5784–5786. 10.1002/nop2.1872. 37246470 PMC10416066

[opn70068-bib-0043] Oyama, Y. , N. Tamiya , M. Kashiwagi , M. Sato , K. Ohwaki , and E. Yano . 2013. “Factors That Allow Elderly Individuals to Stay at Home With Their Families Using the Japanese Long‐Term Care Insurance System.” Geriatrics & Gerontology International 13, no. 3: 764–773. 10.1111/ggi.12002. 23216629

[opn70068-bib-0044] Provencher, V. , L. Clemson , K. Wales , et al. 2020. “Supporting At‐Risk Older Adults Transitioning From Hospital to Home: Who Benefits From an Evidence‐Based Patient‐Centered Discharge Planning Intervention? Post‐Hoc Analysis From a Randomized Trial.” BMC Geriatrics 20, no. 1: 84. 10.1186/s12877-020-1494-3. 32122311 PMC7053102

[opn70068-bib-0045] Purdy, S. , T. Griffin , C. Salisbury , and D. Sharp . 2009. “Ambulatory Care Sensitive Conditions: Terminology and Disease Coding Need To Be More Specific to Aid Policy Makers and Clinicians.” Public Health 123, no. 2: 169–173. 10.1016/j.puhe.2008.11.001. 19144363

[opn70068-bib-0046] Ram, F. S. , J. A. Wedzicha , J. Wright , and M. Greenstone . 2004. “Hospital at Home for Patients With Acute Exacerbations of Chronic Obstructive Pulmonary Disease: Systematic Review of Evidence.” BMJ 329, no. 7461: 315. 10.1136/bmj.38159.650347.55. 15242868 PMC506849

[opn70068-bib-0047] Rudnicka, E. , P. Napierala , A. Podfigurna , B. Meczekalski , R. Smolarczyk , and M. Grymowicz . 2020. “The World Health Organization (WHO) Approach to Healthy Ageing.” Maturitas 139: 6–11. 10.1016/j.maturitas.2020.05.018. 32747042 PMC7250103

[opn70068-bib-0048] Segal, M. , E. Rollins , K. Hodges , and M. Roozeboom . 2014. “Medicare‐Medicaid Eligible Beneficiaries and Potentially Avoidable Hospitalizations.” Medicare & Medicaid Research Review 4, no. 1: 1. 10.5600/mmrr.004.01.b01. PMC4053188 24926414

[opn70068-bib-0049] Shinohara, T. , K. Saida , S. Tanaka , A. Murayama , and D. Higuchi . 2021. “Did the Number of Older Adults With Frailty Increase During the COVID‐19 Pandemic? A Prospective Cohort Study in Japan.” European Geriatric Medicine 12, no. 5: 1085–1089. 10.1007/s41999-021-00523-2. 34081313 PMC8172364

[opn70068-bib-0050] Solberg, L. I. , E. P. Kent , W. E. Ronald , et al. 1990. “The Minnesota Project: A Focused Approach to Ambulatory Quality Assessment.” Inquiry 27, no. 4: 356–367. http://www.jstor.org/stable/29772160. 2148309

[opn70068-bib-0051] Stojak, Z. , J. Jamiolkowski , S. Chlabicz , and L. Marcinowicz . 2019. “Levels of Satisfaction, Workload Stress and Support Amongst Informal Caregivers of Patients Receiving or Not Receiving Long‐Term Home Nursing Care in Poland: A Cross‐Sectional Study.” International Journal of Environmental Research and Public Health 16, no. 7: 1189. 10.3390/ijerph16071189. 30987053 PMC6480023

[opn70068-bib-0052] von Elm, E. , D. G. Altman , M. Egger , et al. 2007. “The Strengthening the Reporting of Observational Studies in Epidemiology (STROBE) Statement: Guidelines for Reporting Observational Studies.” PLoS Medicine 4, no. 10: e296. 10.1371/journal.pmed.0040296. 17941714 PMC2020495

[opn70068-bib-0053] Walsh, E. G. , J. M. Wiener , S. Haber , A. Bragg , M. Freiman , and J. G. Ouslander . 2012. “Potentially Avoidable Hospitalizations of Dually Eligible Medicare and Medicaid Beneficiaries From Nursing Facility and Home‐ and Community‐Based Services Waiver Programs.” Journal of the American Geriatrics Society 60, no. 5: 821–829. 10.1111/j.1532-5415.2012.03920.x. 22458363

[opn70068-bib-0054] Weissman, J. S. , C. Gatsonis , and A. M. Epstein . 1992. “Rates of Avoidable Hospitalization by Insurance Status in Massachusetts and Maryland.” JAMA 268, no. 17: 2388–2394. https://jamanetwork.com/journals/jama/article‐abstract/400938. 1404795

[opn70068-bib-0055] Wolverton, S. , J. Dombrosky , and R. L. Lyman . 2016. “Practical Significance: Ordinal Scale Data and Effect Size in Zooarchaeology.” International Journal of Osteoarchaeology 26, no. 2: 255–265. 10.1002/oa.2416.

[opn70068-bib-0056] Worrall, P. , and T. J. Chaussalet . 2015. “A Structured Review of Long‐Term Care Demand Modelling.” Health Care Management Science 18, no. 2: 173–194. 10.1007/s10729-014-9299-6. 25348171

[opn70068-bib-0057] Xiang, Q. , Y. Li , R. Liang , et al. 2024. “The Geriatric Nutrition Risk Index Is Longitudinally Associated With Incident Sarcopenia: Evidence From a 5‐Year Prospective Cohort.” Aging Clinical and Experimental Research 36, no. 1: 52. 10.1007/s40520-024-02725-7. 38438599 PMC10912133

